# Sentinel Node Biopsy in Early Breast Cancer Patients with Palpable Axillary Node

**DOI:** 10.31557/APJCP.2020.21.6.1631

**Published:** 2020-06

**Authors:** Leyla Shojaee, Sheida Abedinnegad, Nahid Nafisi, Farshad Naghshvar, Gholamali Godazandeh, Siavosh Moradi, Kiarash Shakeri Astani, Yasaman Godazandeh

**Affiliations:** 1 *Department of surgery, Mazandaran University of Medical Sciences, Sari, Iran. *; 2 *Department of Breast Surgery, Iran University of Medical Sciences, Tehran, Iran. *; 3 *Department of Pathology, Mazandaran University of Medical Sciences, Sari, Iran. *; 4 *School of Epidmiology, Mazandaran University of Medical Sciences, Sari, Iran. *; 5 *School of Medicine, Student Research Committee of Mazandaran University of Medical, Sari, Iran. *

**Keywords:** Breast cancer, palpable lymph node, tumor subtype, sentinel lymph node biopsy

## Abstract

**Background:**

Sentinel lymph node biopsy is a reliable method for evaluation of the axillary lymph node status in early stage breast cancer patients with non-palpable lymph nodes. The present study evaluated the status of sentinel and non-sentinel lymph nodes in T1T2 patients with palpable axillary lymph nodes.

**Materials and Methods:**

One hundred and two women with early breast cancer were investigated in this study. Patients were selected for axillary sentinel lymph node biopsy and then surgery .Then the rates of false negative and true positive, and diagnostic accuracy of sentinel lymph nodes biopsy were evaluated. In addition, the hormone receptors status of the tumor was determined through IHC and data was analyzed in SPSS21.

**Results:**

In this study, the mean age of the patients was 49 years, 85% had invasive ductal carcinoma in their pathology reports, 77% were ER/PR positive, 30% HER2 positive and 9.8% triple negative and 69% had KI67<14%. In frozen pathology, 15.7 and 84.3% were sentinel positive and negative, respectively, and in the final pathology, 41 and 58.8% were sentinel positive and negative, respectively. This difference arises from the false negative rate of the frozen pathology, which was about 31.3%. The sensitivity, specificity, and diagnostic accuracy of the frozen section were 24, 90 and 43%, respectively. Lymphovascular invasion is an important effective factor in the involvement of sentinel and non-sentinel lymph nodes. Statistical analysis showed that the probability of sentinel and non-sentinel lymph nodes involvement was higher in receptor positive patients and those with KI67>14% (p<0.002) whereas the rate of involvement was lower in triple negative patients.

**Conclusion:**

Sentinel node biopsy can be used in a significant percentage of breast cancer patients with palpable and reactive axillary lymph nodes.

## Introduction

Axillary lymph nodes receive the lymph from all areas of the breast. Involvement of axillary lymph nodes in breast cancer is an important factor in recurrence and survival of patients with breast cancer, and involvement of a large number of lymph nodes decreases the survival rate (McLaughlin et al., 2008; Yoshihara et al., 2013).

In patients with breast cancer, evaluation of axillary lymph nodes is an important part of staging and treatment of patients. Axillary lymph node dissection is a reliable procedure in staging and an effective method in regional control of the disease (Giuliano et al., 1994). As an effective and reliable method for evaluation of axillary lymph node in patients with early stage breast cancer, sentinel node biopsy has substituted axillary dissection since 1990 (Murray and Given-Wilson, 1997; Veronesi et al., 2010).

It should be noted that axillary lymphadenopathy without a proven malignancy can develop due to benign reactive reactions such as connective tissue disease and skin infections. Therefore, ultrasound can be used extensively for identification of breast and axillary pathologies in breast cancer patients (Patel et al., 2005; Schwab et al., 2010).

Diagnostic accuracy of ultrasound varies and depends on various factors including the operator’s skill and experience, the device type, and the limitation in standards (Ohuchi et al., 2009). According to the results of a study, the sensitivity and specificity of ultrasound for finding a pathologic lymph node are 63.3 and 84.6%, respectively. However, it has a low sensitivity level in detecting lymph node micro-metastases (Rezvani et al., 2018).

Moreover, the relationship between molecular subtypes of breast cancer has been raised as an important factor in the prognosis and survival of patients with breast cancer. Numerous studies have shown that there is a relationship between breast cancer subtypes and axillary lymph node metastasis. One study has proven that luminal A and TNB patients have a lower risk for lymph node metastasis in comparison with luminal B and HER2 patients (Yang et al., 2017).

Anita Mamtani studied the clinicopathologic status and axillary involvement in 701 stage I and stage II breast cancer patients and showed that there was no relationship between axillary lymph node involvement and triple negative breast cancer (Mamtani et al., 2016).

Kaptan Gullben et al. investigated the relationship between tumor subtypes including luminal A, HER2 and luminal/HER2 and the extent of non-sentinel lymph node involvement in 104 breast cancer patients who had undergone axillary lymph node dissection. Univariate and multivariate evaluations showed that tumor size and the extent of lymph node involvement had a significant relationship with non-sentinel lymph node involvement, and that the extent of non-sentinel lymph node involvement was higher in luminal B and HER2 positive patients than in the triple negative patients. They concluded that tumor subtypes were an independent factor in the evaluation of non-sentinel lymph nodes in patients with positive sentinel lymph nodes (Lyman et al., 2014).

The present study performed sentinel lymph node biopsy in patients with T1T2 breast cancer, who had non-pathologic lymph node in clinical examination and ultrasound, to evaluate the pathologic results of axillary sentinel and non-sentinel lymph nodes and to determine the relationship between tumor subtype and the extent of involvement. In addition, it tried to predict the possibility of axillary lymph node involvement based on the ultrasound results and tumor subtype. According to the results of this study, could be sentinel biopsy used in patients with palpable and normal lymph node in ultrasound? Could they enjoy the benefit of sentinel biopsy and avoid axillary dissection merely due to palpability of the lymph nodes?

## Materials and Methods

This study was performed on 102 patients with breast cancer who visited Imam Khomeini Hospital in Sari, Iran, from 2016 to 2018. The study was approved by and registered in the Cancer Research Center of Mazandaran University of Medical Sciences. Patients with a pathology report of invasive breast cancer and tumor size of less than 5 cm whose axillary lymph nodes seemed non-pathologic in clinical examination and ultrasound were selected for sentinel lymph node biopsy. Patients’ demographic information and their pathologic reports before and after breast surgery as well as their axillary pathology reports were collected and analyzed.

The exclusion criteria were advanced breast cancer (stages III and IV), primary tumor size of larger than 5 cm, unwilling to participate in the study, DCIS patients and patients who received chemotherapy. Moreover, patients in whom sentinel lymph node was not detected during surgery, and axillary dissection was performed on them, were excluded from the study. All patients underwent bilateral axillary ultrasound by an experienced radiologist before the surgery and were selected for sentinel biopsy if the axillary lymph node was reactive and non-pathologic. A few hours before surgery, the radioisotope (technetium 99) was injected by a nuclear medicine specialist into the peri areolar or peritumoral area and the patients were transferred to the operating room after lymphoscintigraphy and identification of the sentinel lymph node. The sentinel lymph nodes were identified before surgery using a gamma scanner and lymph nodes with more than 10% maximum radiation absorption in the injection site were removed and sent for frozen pathology to the Pathology Department. In the Pathology Department, lymph nodes smaller than 1 cm were incised with one incision and those larger than 2 cm with 2. Half of the lymph node was used for frozen section pathology. It was frozen at -30°C, stained with H&E, and microscopically examined by an experienced pathologist. After the initial evaluation, it was fixed in formalin for definitive pathologic evaluation. The remaining half of the lymph node was fixed in formalin for definitive pathology examination. If the frozen pathology report indicated that the lymph node was metastatic, axillary dissection was performed in the same stage. However, if the lymph node was negative and the final pathology report indicated metastatic involvement, two weeks after the first surgery a second delayed axillary surgery was performed and the lymph node was dissected. The final results concerning the numbers of involved sentinel and non-sentinel lymph nodes in patients who underwent the primary or delayed dissection of the axillary lymph nodes were studied and factors affecting the probability of involvement of axillary sentinel and non-sentinel lymph nodes were analyzed. In addition, immunohistochemistry (IHC) study was conducted on tumors, and the statuses of the estrogen, progesterone, HER2, and KI67 receptors, and of the triple negative receptors, were determined.

*Statistical analysis*

First, the type of data distribution was examined by plotting the histogram and performing the Kolmogorov-Smirnov and/or Shapiro-Wilk test. Then the quantitative data were described through the calculation of mean, standard deviation, median and interquartile range. The qualitative data were described through calculation of frequencies and percentages. The relationship between qualitative variables was examined through Chi-square and/or Fischer exact tests. In addition, the odds ratio for each of the molecular subtypes in involvement of sentinel and non-sentinel lymph nodes was calculated by performing logistic regression.

The data were described and analyzed using IBM SPSS 21. For all the statistical tests, the bilateral p-value <0.05 was considered significant. 

## Results

One hundred and two women with a mean age of 49 years, range 30-79 years, having breast cancer with tumor size smaller than 4 cm and lymph nodes non-pathologic in ultrasound underwent axillary sentinel node biopsy. The histopathology was invasive ductal cancer in 97% of the tumors. All the patients had breast conserving surgery. A radioisotope (technetium 99) was injected preoperatively, the axillary sentinel lymph node was biopsied during the surgery and the frozen section was performed. The results of the frozen pathology was positive in 9.8% of the cases and these patients underwent axillary lymph node dissection. 53.3%of the patients had negative lymph node and hence did not undergo dissection during the surgery. Thirty-one point three of the results were false negative and 5.8%false positive. According to the final pathology report, 58.8% of the patients (including true negative sentinel node patients plus false positive sentinel node patients) had no lymph node involvement. In the final pathology report, 41.1% of lymph nodes (including true positive sentinel node patients plus false negative sentinel node patients) were involved (Table 1). 

**Figure 1:**
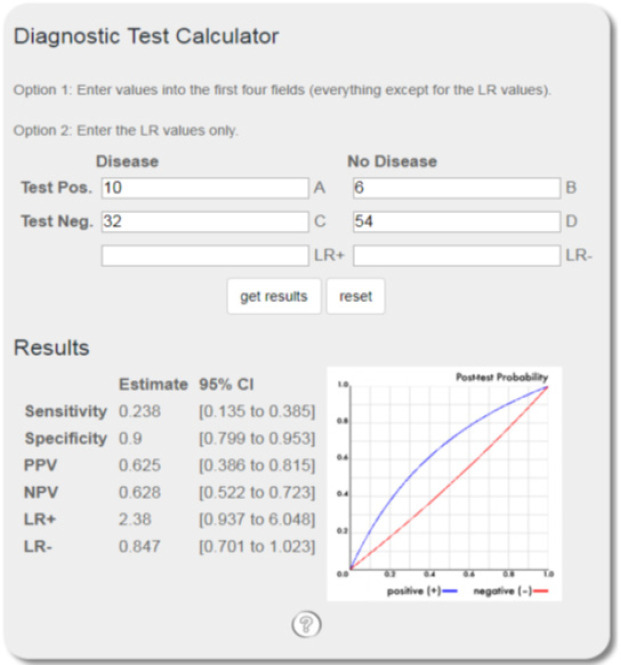
Results Related to Analysis of Diagnostic Accuracy of the Frozen Report in Comparison with the Golden Standard of the Final Pathology Report Performed Online with Diagnostic Test Calculator (https://ebm-tools.knowledgetranslation.net/calculator/diagnostic/). The frozen section had the sensitivity and specificity of 24 and 90%, respectively, and the general diagnostic accuracy 0.431. The reason for the low sensitivity was the high number of false negative cases

All patients with a false negative frozen report underwent delayed axillary dissection. The sensitivity, specificity, and diagnostic accuracy of the frozen section were 24, 94, and 43%, respectively (Figure 1).

IHC study was conducted on the tumors and the statuses of hormone receptors including estrogen, progesterone, HER2, KI67and of triple negative receptors were determined. Seventy-five-point five percent were estrogen receptor positive, 70% progesterone receptor positive, 31.4% HER2 positive, 67.6% KI67<14% and 14.7% triple negative. In this analysis, correlations between the positive status of hormone receptors and positive lymph node in the frozen section and the final pathology report were investigated. The results of Kendall’s tau-b coefficient and Spearman’s rho rank correlation coefficient are presented in Table 2. This Table shows that there was a statistically significant correlation between the positive status of ER and PR and involvement of the sentinel lymph node in final frozen pathology. In addition, the correlation between the status of KI67>14% and involvement of the sentinel lymph node in the frozen section was statistically significant.

The relationships between hormone receptors with axillary sentinel and non-sentinel lymph nodes were investigated in the patients. The results showed that the likelihood of either sentinel or non-sentinel lymph nodes involvement was higher in the estrogen and progesterone positive patients and lower in the triple negative patients. In addition, lymphovasuclar invasion was an effective factor in the involvement of axillary sentinel and non-sentinel lymph nodes (Table 2).

**Table 1:** The Status of Axillary Sentinel Lymph Node in the Frozen and Final Pathology

	Frequency	Percentage
TP	10	9.8
TN	54	53.0
FP	6	5.8
FN	32	31.3

**Table 2:** Relationship between Tumor Receptors and Sentinel and Non-sentinel Axillary Lymph Nodes

Receptor positive	SLN+ in frozen section				SLN+ in final pathologic report			
	Kendall’s tau-b		Spearman’s Rho		Kendall’s tau-b		Spearman’s Rho	
	Coefficient	p-value	Coefficient	p-value	Coefficient	p-value	Coefficient	p-value
ER	0.183	0.066	0.183	0.065	0.199	0.046	0.199	0.045
PR	0.168	0.092	0.168	0.092	0.293	0.003	0.293	0.003
HER2								
Ki67>14%	0.267	0.007	0.267	0.006	-0.029	0.771	-0.029	0.773
Triple negative							0.235	0.018
LVI			0.250	0.927			0.221	0.001

**Table 3:** Presents the Result of Ordinal Regression for Evaluation of the Relationship between the Nodes Ratio (<25%, >25%) and the Different Receptors. As Can be Seen, the Receptor Status Cannot Predict NR

		parameter Estimates					95% Confidence interval	
		Estimate	Std.Error	Wald	df	Sig.	Lower Bound	Upper Bound
Threshold	[Nod Ratio Stat =1]	1.87	0.732	6.53	1	0.011	0.436	3.305
	[ER=1]	0.663	1.128	0.345	1	0.557	-1.548	2.875
	[ER=2]	0^a^	.	.	0	.	.	.
	[PR=1]	-0.137	1.038	0.017	1	0.895	-2.172	1.898
	[PR=2]	0^a^	.	.	0	.	.	.
Location								
	[HER2=1]	0.413	0.53	0.607	1	0.436	-0.626	1.452
	[HER2=2]	0^a^	.	.	0	.	.	.
	[K167=1]	0.049	0.532	0.009	1	0.926	-0.994	1.092
	[K167=2]	0^a^	.	.	0	.	.	.

Link function, Legit; a.This parameter is set to zero because it is redundant.

## Discussion

Evaluation of axillary lymph nodes is an important part of breast cancer staging. It can be performed through clinical examination, ultrasound, the FNA procedure or a core needle biopsy. Sentinel node biopsy and axillary dissection are then carried out. Since the introduction of Halsted’s radical mastectomy, axillary dissection has been a successful method for local control and treatment of patients (Gülben et al., 2014). Sentinel lymph node biopsy is a method that can substitute axillary lymph node dissection in patients with early stage breast cancer who have non-palpable lymph nodes. It is currently a standard method with fewer complications for evaluation of lymph node involvement in this group of patients (Rao et al., 2013; Lyman et al., 2014).

Results of studies in recent years have changed the management of patients with low tumor burden in axillary lymph node involvement so that treatments have increasingly tended to be less invasive due to the early and delayed complications of axillary treatment. For example, the ZOO11 trial recommended that axillary dissection be omitted in patients who were candidates for breast conserving surgery and had less than 2 lymph nodes involved in the sentinel lymph node evaluation (Giuliano et al., 2011)

Evaluation of axillary lymph nodes in early stage breast cancer patients with clinically negative lymph node is performed through sentinel node biopsy (Keshtgar and Ell, 2002; Veronesi et al., 2003; Zhou et al., 2011). However, lymphadenopathy may have reactive causes such as inflammatory diseases and skin infections, and preoperative ultrasound can report the probability of pathologic or reactive causes of lymph nodes based on characteristics of lymph nodes such as their shape (oval, round) and cortex thickness (Lim et al., 2004; Muttarak et al., 2004). However, ultrasound has limited diagnostic power and cannot identify micro-metastases (Chu et al., 1999).

Sentinel node biopsy is the standard procedure in patients with clinically negative lymph nodes, and in cases of positive sentinel node it is followed by dissection. However, 40-70% of these patients have no metastasis in the non-sentinel lymph nodes (Chu et al., 1999; Hwang et al., 2003; Nos et al., 2003)and axillary dissection has the negligible therapeutic effect for them (Nos et al., 2003; Moghaddam et al., 2010).

Breast cancer is a heterogeneous tumor with different molecular subtypes, clinical manifestations, and response to treatment. Numerous studies have investigated the relationship between breast cancer subtypes and axillary lymph node involvement. Geok Hoon Lim et al. tried to identify patients with high node burden to avoid sentinel node biopsy for them. They investigated 1298 T1T2N0 patients and found that sentinel node biopsy could be avoided and axillary dissection be performed in patients with ≤4 abnormal lymph nodes in ultrasound (Hirko et al., 2013; Lim et al., 2019)

Another study revealed that there was a relationship between breast cancer subtypes and axillary metastasis, and that luminal A and TNB patients had a lower risk for node involvement than luminal B and HER2 patients (Yang et al., 2017).

In addition, a study in 2014 showed that luminal B patients (either HER2+ or HER2-) had a strong relationship with node involvement, and that triple positive and triple negative breast cancer exhibited the highest and lowest probability of axillary lymph node involvement, respectively (Si et al., 2014).

Öz et al., (2016) studied 110 sentinel node positive patients who underwent axillary dissection 101 of whom had up to 2 involved lymph nodes and 32 showed non-sentinel lymph node involvement. Univariate and multivariate analyses indicated that lymphovascular invasion and positive ER and PR, HER2 and KI67>14% receptors were associated with higher involvement of non-sentinel lymph nodes.

In a study by Ahmed (2016), HER2 was introduced as a potent prognostic factor in lymph node metastasis in breast cancer. In this study, the primary breast cancer tumors of 317 patients were investigated for estrogen, progesterone and HER2 receptors using IHC staining. A relationship was found between these markers and axillary lymph node metastasis by employing statistical methods. They noticed that ER, PR, and HER2 were expressed in 35.7, 73.2 and 19.9% of the tumors, respectively. In addition, the tumor size and grade had a direct relationship with HER2 and inverse relationships with PR and ER. Univariate analysis revealed that HER2 had a direct relationship with axillary lymph node metastasis.

This study concluded that lymph nodes were palpable in some breast cancer patients but they were motile, small, and soft and non-pathologic and reactive in ultrasound (nodes were smaller than 2 cm, normal cortex thickness or were reactive and oval-shaped with normal fat), lymph node involvement was less likely in them, and they benefited from sentinel node biopsy. These patients were candidates for sentinel lymph node biopsy and, if the biopsy result was positive, they underwent axillary dissection. The extent of involvement of axillary sentinel and non-sentinel lymph nodes was then investigated and the relationship between the status of the receptors including ER, PR, HER2, TN, and KI67 and the probability of lymph node involvement was evaluated to find predictive factors that could predict the probability of lymph node metastasis based on ultrasound findings and on the status of tumor receptors.

More than 50 percent (53%) of the patients were with negative sentinel lymph nodes that were not dissected and 9.8%% of the patients were sentinel lymph node positive in frozen section. In some studies on rate of false negative sentinel biopsy in clinically lymph node negative patients the reported rate of false negative frozen was 10 to 60%, but most of such studies have reported rates of 15-20% (Nos et al., 2003; Moghaddam et al., 2010; Hirko et al., 2013). In the present research, the rates of false negative sentinel and false positive sentinel were 31.3 and 5.8%, respectively, and the diagnostic accuracy of frozen section was 43%. Forty seven percent of patients underwent axillary lymph node dissection in 21% of whom the extent of tumor burden (Node ratio or NR, which is the ratio of the number of metastatic lymph nodes to the total number of dissected) was ≤ 25% and in 79% it was more than 25% (Table 3). In fact, the majority of the patients had micro-metastatic involvement with less than 3 involved nodes. The probability of involvement was higher in estrogen and progesterone positive patients (p<0.003). In addition, the likelihood of non-sentinel node involvement was higher in patients with KI67>14% (p<0.006) and also in patients with lymphovascular invasion (p<0.001), but the probability of non-sentinel node involvement was lower in triple negative patients (p<0.018). No specific and statistically strong relationship was found between nodal ratio and receptor expression.

The results of many studies on the relationship between breast cancer subtypes and axillary lymph node involvement have been contradictory. Some have reported higher likelihood of lymph node involvement in receptor positive patients whereas others have reported higher probability of lymph node involvement in triple negative patients. Therefore, this issue needs further research.

The present study evaluated the relationship between hormone receptors and involvement of axillary lymph nodes, and then used this predictive power for selecting T1T2 patients (who had palpable lymph nodes but were clinically non-pathologic and had no abnormal ultrasound) as candidates for sentinel biopsy instead of lymph node biopsy using COR or FNA. Finally, it tried to determine how likely it was to find non-sentinel lymph node involvement in the patients with false negative sentinel results.

Our results indicated that, if this group of patients underwent sentinel biopsy, the rates of false negative and false positive would be higher. However, according to recent trials such as ZOO11 and the AMAROSE, the likelihood of non-sentinel lymph node involvement is lower in patients with less than 3 involved nodes in the final pathology report who also have the receptor negative criterion (K167<14) and are LVI negative. Therefore, it is possible to avoid axilla dissection in these patients and to locally control axilla by using radiotherapy.

Study of the relationship between tumor subtypes with estrogen and progesterone positive receptors and patients with KI67>14% showed that the likelihood of sentinel and non-sentinel lymph node involvement was higher, but this relationship lacks considerable predictive power because of the small sample size. However, our study showed that the majority of patients who were deprived of sentinel evaluation merely based on clinically palpable lymph node had no pathologic lymph node involvement in the sentinel nodes or in the non-sentinel nodes. Therefore, they suffered from complications of axillary dissection.

We suggest that a study with a larger sample size be conducted to find a way for identifying those patients who are appropriate candidates for sentinel biopsy in whom there is less likelihood of lymph node involvement.
